# The role of lactose in weanling pig nutrition: a literature and meta-analysis review

**DOI:** 10.1186/s40104-020-00522-6

**Published:** 2021-01-11

**Authors:** Jinbiao Zhao, Zeyu Zhang, Shuai Zhang, Greg Page, Neil W. Jaworski

**Affiliations:** 1 State Key Laboratory of Animal Nutrition, College of Animal Science and Technology, China Agricultural University, No. 2 Yuanmingyuan West Road, Beijing, 100193 China; 2 Trouw Nutrition Innovation, Stationsstraat 77, 3800AG Amersfoort, Netherlands

**Keywords:** Growth performance, Gut health, Gut microbiota, Lactose, Lactose equivalents, Weanling pig

## Abstract

Lactose plays a crucial role in the growth performance of pigs at weaning because it is a palatable and easily digestible energy source that eases the transition from milk to solid feed. However, the digestibility of lactose declines after weaning due to a reduction in endogenous lactase activity in piglets. As a result, some lactose may be fermented in the gastrointestinal tract of pigs. Fermentation of lactose by intestinal microbiota yields lactic acid and volatile fatty acids, which may positively regulate the intestinal environment and microbiome, resulting in improved gastrointestinal health of weanling pigs. We hypothesize that the prebiotic effect of lactose may play a larger role in weanling pig nutrition as the global feed industry strives to reduce antibiotic usage and pharmacological levels of zinc oxide and supra-nutritional levels of copper. Evidence presented in this review indicates that high dietary lactose improves growth performance of piglets, as well as the growth of beneficial bacteria, particularly *Lactobacillus*, with the positive effects being more pronounced in the first 2 weeks after weaning. However, the risk of post-weaning diarrhea may increase as pigs get older due to reduced lactase activity, high dietary lactose concentrations, and larger feed intakes, all of which may lead to excessive lactose fermentation in the intestine of the pig. Therefore, dietary lactose levels exert different effects on growth performance and gastrointestinal physiological functions in different feeding phases of weanling pigs. However, no formal recommendation of lactose for weanling pigs has been reported. A meta-analysis approach was used to determine that diets fed to swine should include 20%, 15%, and 0 lactose from d 0–7, d 7–14, and d 14–35 post-weaning, respectively. However, sustainable swine production demands that economics must also be taken into account as lactose and lactose containing ingredients are expensive. Therefore, alternatives to lactose, so called “lactose equivalents” have also been studied in an effort to decrease feed cost while maintaining piglet performance with lower dietary lactose inclusions. In summary, the present review investigated dose-response effects of dietary lactose supplementation to exert positive responses and begin to elucidate its mechanisms of action in post-weaning pig diets. The results may help to replace some or all lactose in the diet of weanling pigs, while improving production economics given the high cost of lactose and availability in some swine production markets.

## Introduction

Lactose is a disaccharide present in milk and is the main carbohydrate source for infant mammals. Lactose is digested by lactase into glucose and galactose in the small intestine providing readily absorbable energy for young mammals [1]. A portion of dietary lactose is fermented by bacteria, like *Lactobacillus,* in the stomach, producing lactic acid and minimal quantities of acetate [2], which maintain gastric acidity in suckling piglets [3]. Endogenous lactase activity in pigs sharply declines at weaning [4]. Piglets are weaned, on average in the global swine industry, 3 to 4 weeks after birth, which results in a decreased capacity to digest high levels of lactose [4]. Recent research in humans suggests the decrease in lactase activity to be independent of the amount of lactose and the duration of lactose intake in the diet [5]. Therefore, a certain amount of lactose may reach the large intestine of the pig and be a substrate for microbial fermentation to produce lactic acid and volatile fatty acids (VFA), which have been reported to be beneficial to gut health and host metabolism [6, 7].

The prebiotic effect of lactose may now play a larger role in weanling pig nutrition as the global feed industry strives to reduce antibiotic usage, pharmacological levels of zinc oxide (ZnO), and supra-nutritional levels of Cu. Recent studies, especially in the human infant and lactose intolerant realms, have focused on exploring the prebiotic effect of lactose. Lactose has been described as a prebiotic that exerts health benefits through selective stimulation of intestinal bifidobacteria and lactobacilli in adults with impaired lactose digestion and without a history of gastrointestinal disease [8, 9]. Some studies reported that the prebiotic health benefits of lactose were not only associated with intestinal microbial composition, but also related to many different microbial metabolites, one of which is butyrate, which is an important energy source for the colonic mucosa and has anti-inflammatory effects amongst several other health benefits [10, 11]. Moreover, acid production in the gastrointestinal tract has been shown to modulate the composition of gut microbiota by decreasing intestinal pH, thereby reducing growth and proliferation of some intestinal pathogens [12]. However, an explicit requirement for dietary lactose in weanling piglets is not available [13]. Suckling and weanling pigs most probably do have a specific requirement for lactose as they consume a milk-based diet from the sow and transition onto a lower lactose creep feed, to an even lower lactose concentrated solid feed after weaning. On the other hand, excessive intake and over-fermentation of lactose may lead to intolerance symptoms in humans resulting in diarrhea and this may also occur in pigs as their gastrointestinal system is very similar to humans [5]. Therefore, dietary lactose supplementation in weanling pig nutrition may be a balancing act between providing an easily digestible energy source and an easily fermentable prebiotic source. This review investigated mechanisms of action of lactose when included in post-weaning swine diets with the aim to disclose nutritional and prebiotic effects and the optimal concentration of lactose in weanling pig diets.

## Definition of lactose

Lactose is a disaccharide consisting of α-*D*-glucose and β-*D*-galactose, which is chemically formed as O-β-*D*-galactopyranosyl-(1-4)-β-glucose [14]. Carbon 1 of the glucose moiety is anomeric because it carries a hydroxyl group which is free to lie above or below the plane of the ring. This hydroxyl group is responsible for the existence of α- and β-lactose forms, which can be interconverted in aqueous solution under different processing temperatures. α-lactose crystallizes from super-saturated solutions at temperatures below 93.5 °C to produce a variety of crystal shapes, but above 93.5 °C, β-lactose is transformed as an uneven-sided diamond. β-lactose is more easily dissolved in water and sweeter compared to α-lactose. Therefore, β-lactose may improve pig feed intake to a greater extent compared with α-lactose, but no studies have tested this hypothesis. α-lactose is primarily used in the pharmaceutical industry as a carrier for pill manufacturing because of its stability and good storage properties [15]. However, anomeric composition of the wide range of commercially available lactose sources has not been investigated and the development of rapid methods for distinguishing the anomeric composition of lactose products may be warranted.

## Lactose in sow milk

**Fig. 1:**
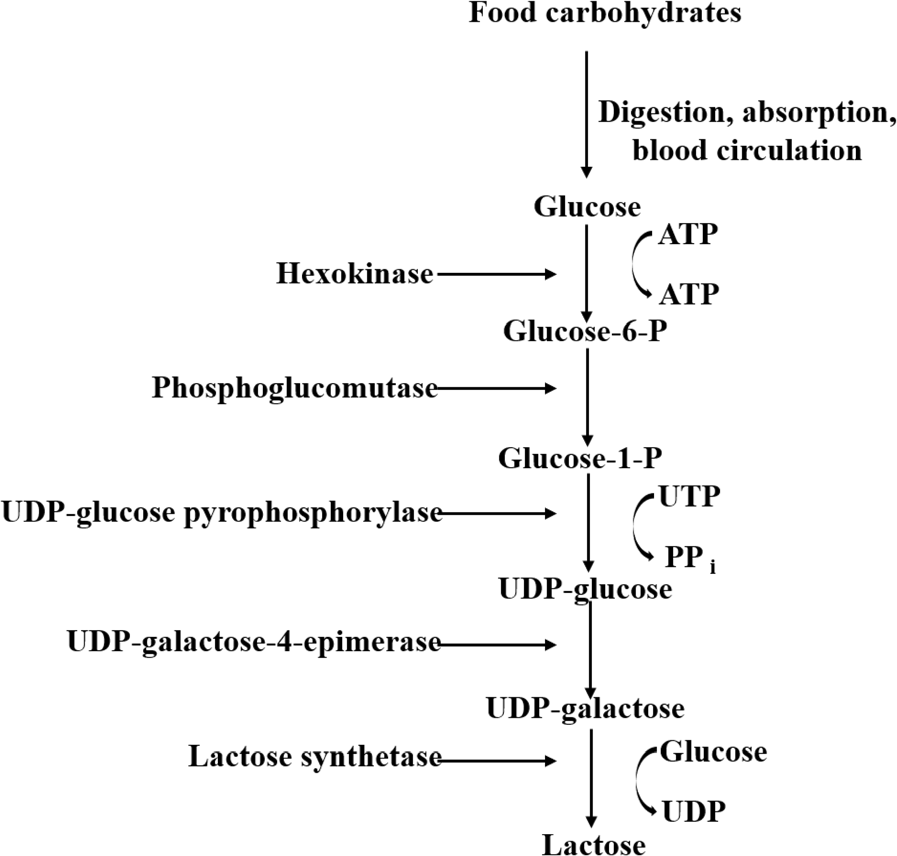
The biosynthesis of lactose in the mammary gland of sows (adapted from Blackburn et al. [16])

The disaccharide lactose occurs almost exclusively in the milk of mammals. Glucose is the sole precursor of lactose in animals and the synthesis of lactose from glucose requires a number of enzyme-mediated steps (Fig. 1). Importantly, glucose must be derived from nutrient digestion due to a lack of glucose-6-phosphatase in the sow, then be readily absorbed from the small intestine into the bloodstream, and then flow into mammary arterial blood [16].

Lactose concentration in milk differs among animal species. Human milk contains 60–70 g/L lactose in addition to 12–15 g/L milk oligosaccharides. While the milk of goat, sheep, and cow, all contain between 40 and 50 g/L lactose and only a small fraction of milk oligosaccharides [17]. More similar to human milk, sow colostrum and milk contain 40 g/L and 60 g/L of lactose, respectively [18, 19]. The milk oligosaccharide content of sow milk is as high as 23 g/L in colostrum and rapidly decreases during lactation to values as low as 5 to 10 g/L in late lactation. The primary porcine milk oligosaccharides (MOs) are fucosylated-MOs, sialylated-MOs, and neutral-MOs and these act as prebiotics for suckling pigs [20].

Milk and lactose yield in sows were reported to be associated with type of diet, lactation environment, suckling frequency of piglets, and health status [21]. Temperatures on either side of the thermoneutral zone, in addition to high relative humidity, have been shown to decrease milk lactose yield in lactating sows [22]. Zhao et al. [23] reported that the increased proportion of valine to lysine from 0.72% up to 1.01% in the diet of sows from d 29 prepartum to d 21 postpartum increased lactose concentration of sow colostrum in a hot and humid environment. Dunshea et al. [24] infused a combination of insulin (11 mU/kg per hour) and dextrose (50%) into the anterior vena cava of sows using silastic catheters during mid (d 5–10) and late (d 17–22) lactation, and this resulted in increased concentration of lactose in milk. Crude protein concentrations of 16% or 23% supplied by varying quantities of fish meal, blood meal and branched-chain amino acids in diets fed to sows throughout lactation had no effect on lactose concentration in milk [24]. Dourmad et al. [25] also observed that lactating sows fed diets containing different concentrations of crude protein (15.5% or 17%) or lysine (0.66%, 0.77%, or 0.87%) had no effect on sow milk lactose content. Other reports have also indicated that sows fed diets containing higher levels of branched-chain amino acids, tryptophan, or lysine had no effect on sow milk lactose concentration [18, 26]. Furthermore, Yang et al. [27] determined that sows fed 13.7, 13.9, or 14.2 MJ metabolizable energy (ME) per kg during late gestation and lactation did not affect lactose content in sow milk. The supplementation of 0.8% or 1.2% potassium diformate to diets fed from mating until the next mating, or 0.2% betaine from 5 d before the expected date of farrowing until the end of lactation had no effect on the lactose concentration of sow milk or colostrum [28, 29]. Overall, literature evidences suggest that sow milk and colostrum lactose concentrations are difficult to change through dietary macronutrient density or addition of different feed additives in sow diets.

## Digestion and absorption of lactose in suckling and weanling pigs

Piglets are born with high intestinal lactase activity and are generally able to fully digest dietary lactose. Lactase belongs to a group of intestinal disaccharidases located on the brush border of the small intestine. Normally, the activity of lactase is highest in the proximal part of the jejunum and progressively declines towards the ileum [30]. Lactase hydrolyses lactose into the monosaccharide sugars glucose and galactose, which are then available for absorption [1]. Lactase gene expression is initiated before the birth of piglets, remains high during nursing, and then sharply declines after weaning [30]. Feeding neonatal piglets for 6 h with milk replacer, instead of porcine colostrum, was shown to significantly decrease total lactase activity by 25% [31]. De Vos et al. [32] reported feeding colostrum and milk rapidly induced an increase in lactase activity (7 U/g of tissue) compared with those fed a formulated diet (3 U/g of tissue), especially during the first week post-farrow. However, a significant decline in lactase activity in the small intestine of weanling pigs was observed between 3 and 5 weeks of age despite the presence of considerable quantities (19%) of dietary lactose [4], which suggests that mucosal lactase activity is independent of the amount of lactose and the duration of lactose intake in the diet [5]. Weaning, therefore, appears to be the major cause of reduced lactase activity and this is independent of lactose concentration in the diet and duration of feeding lactose containing diets. This implies that some lactose may escape digestion due to reduced lactase activity after weaning and this may result in excessive fermentation of lactose by gut microbiota and exacerbate enteric diseases resulting in post-weaning diarrhea. Indeed, weanling pigs fed a diet containing 29.5% lactose from d 14–21 post-wean resulted in increased diarrhea compared with pigs fed a diet containing 17.5% lactose due to reduced lactase activity and excessive lactose fermentation in the large intestine [33]. It is hypothesized that diarrhea induced by a high concentration of lactose in diets may be a result of poor microbial adaptation to a large flow of easily and, most probably, rapidly fermentable lactose.

In newborn piglets, acid secretion is low in the stomach. The principal source of stomach acidity is from bacterial fermentation of lactose into lactic acid as well as acetate, which activates endogenous enzymes and gut microbiota and improves gut health of suckling pigs [34]. Some lactose will escape bacterial fermentation in the stomach and flow into the small intestine to be enzymatically hydrolyzed by endogenous lactase to be used as an energy source [35, 36]. In addition to endogenous lactase activity, many bacteria in the gastrointestinal tract of pigs express β-galactosidase activity that allow microbes to utilize lactose and produce lactic acid and VFA through fermentation [37]. This is most probably beneficial in the foregut, where bifidobacteria and *Lactobacillus* dominate, especially in a healthy, non-challenged environment. However, this could be detrimental in a disease challenged gut as *E. coli* and streptococci also possess β-galactosidase.

The adult pig, on the other hand, has limited lactase activity in the small intestine and, therefore, lactose may be a significant substrate for microbial fermentation in the small and large intestine. Pierce et al. [38] reported that butyric acid concentration linearly increased and a ratio of acetic acid to propionic acid linearly decreased in the feces of growing-finishing pigs fed diets containing 0 up to 12% lactose. These results indicate that a certain amount of lactose entered the hindgut and was fermented causing shifts in microbial metabolite production. Williams et al. [39] reported that enteric health was improved when the fermentation of lactose occurs in the large intestine. Lactose can be fermented to generate lactic acid and VFA by gut bacteria in the hindgut of pigs [33], which improve intestinal health by modifying gut microbiota and induce the expression of porcine host defense peptides, such as pBD2, pBD3 and pEP2C [6, 7]. Previous studies have shown improved insulin sensitivity through VFA-mediated glucose homeostasis by activating G protein-coupled receptors 41 and 43 and stimulating enteroendocrine L-cells to produce glucagon-like peptide 1 and peptide YY [40, 41]. Overall, lactic acid and VFA produced by intestinal microbiota to ferment lactose play a crucial role in improving intestinal health of piglets [42].

## Effect of lactose level on growth performance and diarrhea

Dietary lactose supplementation was shown to improve the growth performance of post-weaned pigs [43]. The positive effect of dietary lactose on weanling piglets was mainly attributed to its sweetness resulting in improved diet palatability observed through increased average daily feed intake (ADFI). In addition, Partridge and Gill [44] attributed some of the improved growth performance to reduced stomach pH post-weaning through production of lactic acid and VFA from lactose fermentation. Furthermore, a lower pH in the stomach inhibits pathogen growth and improves protein digestion [45]. The inclusion of 25% lactose improved feed intake and weight gain of piglets weaned at 3 to 4 weeks compared with piglets fed a diet containing only 15% lactose from d 0–7 after weaning [46–48]. A linear increase in average daily gain (ADG) and a quadratic increase in feed efficiency (G:F) were observed during the second week after weaning in pigs weaned at 24 days old with an average initial body weight (BW) of 7.1 kg and fed diets containing 6.5%, 17.0%, or 28.0% lactose [49]. In a follow-up study, pigs with an average initial BW of 6 kg (weaned at 24 days old) and fed a diet containing 29.5% lactose had greater ADFI and ADG than those fed a diet with 17.5% lactose during the first week after weaning [33]. On the other hand, piglets (weaned at 23 days old and an average initial BW of 6.5 kg) fed a diet containing 35.5% lactose had lower ADG compared with pigs fed a diet with 22.5% lactose in the first 2 weeks post-wean [50]. Pierce et al. [33] reported that piglets fed a diet with 29.5% lactose had lower diarrhea incidence in the first week after weaning, but showed higher diarrhea incidence in the second and third weeks compared with pigs fed a 17.5% lactose diet. These results indicate that a level of 29.5% lactose in the diet may have exceeded the lactase activity of pigs between 2 and 3 weeks post-weaning, resulting in excessive fermentation of lactose and incidence of diarrhea. Bertol et al. [51] found lactose levels up to 21% in diets for piglets weaned at 21 d of age linearly increased the ADFI and ADG of pigs in the first 14 days post-weaning. Mahan et al. [52] reported piglets with an average initial BW of 6.3 kg, weaned at 19 d of age, and fed diets containing 10% to 35% lactose in the first 2 weeks post-weaning, showed a linear increase in ADG and improved G:F in the first week. Increasing the dietary lactose levels (0, 10%, and 15%) in the first 2 weeks after weaning enhanced growth performance of piglets (mean initial BW of 6.4 kg and average age of 18 d) fed using a liquid feeding system, but this advantage was not maintained after 2 weeks post-wean [53]. Similarly, weanling pigs with initial BW of 6 kg (weaned at 20 days old) that received a diet with 20% lactose in the first 2 weeks had greater growth performance, although no positive effects were observed after 3 weeks post-wean when pigs were fed a diet containing 15% lactose [54]. Taken together, these results indicate that the positive effect of lactose on performance of weanling piglets mainly exist in the first 2 weeks after weaning. This is in general alignment with the observed declines in endogenous lactase activity noted earlier. Interestingly, piglets fed high lactose from 4 d of age to weaning showed greater growth performance in the nursery period, which persisted throughout the finisher and ended in greater slaughter weights, as well as hot and cold carcass weights, compared with a low lactose diet [55]. Piglets weaned at 21 or 28 d of age, and fed a diet with 25% lactose from d 0–7 post-wean, had a positive response on subsequent performance in the whole nursery period in comparison with piglets fed 15% lactose [48]. These results indicate that high dietary lactose in the first 2 weeks post-weaning may be beneficial to performance of pigs in subsequent growth phases.

On the other hand, there have been studies which reported no or a negative response to increased lactose levels in diets fed to weanling pigs. Pollmann et al. [56] found adding 10% lactose to the diet did not improve growth performance of piglets (initial BW of 6 kg) assessed weekly until 28 d post weaning. Furthermore, no growth performance response was observed when pigs (initial BW of 7.05 kg and weaned at 24 days old) were fed diets containing 10% or 20% lactose in the first 2 weeks post-wean [57]. Lactose included in diets at 0, 4%, 8% and 12% did not affect ADG, ADFI, and G:F of weanling pigs (initial BW of 6.1 kg and weaned at 21 d of age) in the first 2 weeks after weaning [58].

The inconsistent observations reported in growth performance of pigs fed diets with higher lactose concentrations may be associated with feeding environment, dietary ingredient and nutrient composition, and weaning age. But, most importantly, weaning stress impacts post-weaning pig performance to a great extent and is the most probable cause for variation in pig response to different levels of dietary lactose. Mahan et al. [52] recommended that diets fed to pigs (weaned at 19 days old and average initial BW of 6.4 kg) from d 0–7, d 7–21, and d 21–35 post-wean should contain 25–30%, 15–20%, and 10–15% lactose, respectively. Cromwell et al. [59] recommended that dietary lactose should be 20% in diets fed from d 0–7 post-wean, 15% in diets fed from d 7–14 post-wean, and 7.5% in diets fed from d 14–30 post-wean.

## Model to predict performance responses of weanling pigs fed different concentrations of dietary lactose using a meta-regression technique

**Fig. 2:**
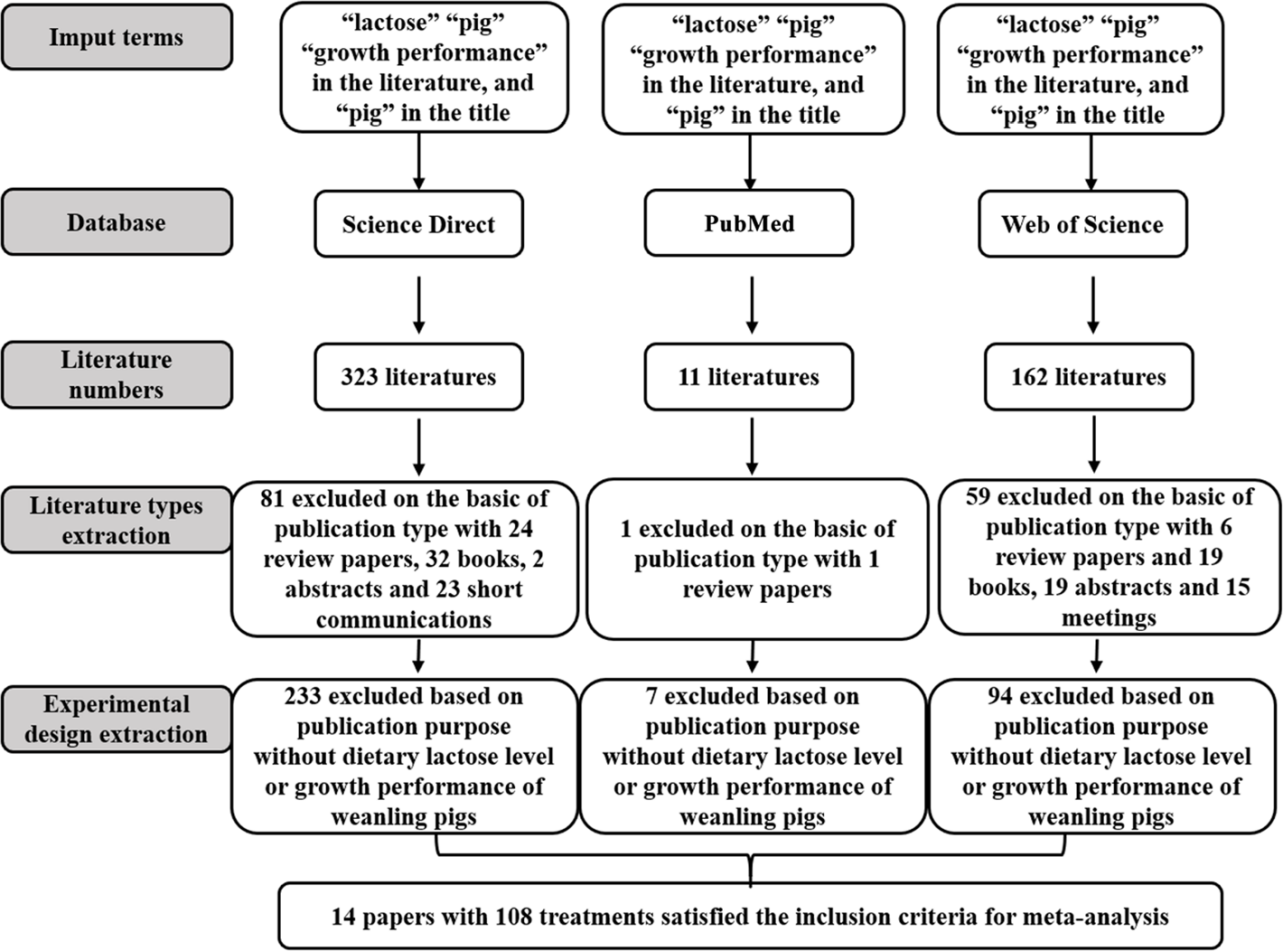
Flow chart of the literature search, screening and data extraction procedures

Growth performance (ADFI, ADG, and G:F) of weanling pigs fed diets containing different levels of lactose were modelled using a meta-regression technique in the present review. First, a database was constructed from published literature which reported growth performance of weanling pigs fed different levels of dietary lactose. Studies were from published articles in scientific journals indexed in public data search generators (PubMed, Web of Science and Science Direct). The literature search, screening, and data extraction processes are shown in Fig. 2. The inclusion criteria for studies were as follows: 1) peer-reviewed and published in the English language from 1990 to 2019; 2) lactose study on weanling pigs; 3) dietary lactose levels as the sole source of variation. A total of 14 published studies were used to create response models that predicted the performance of weanling pigs fed varying levels of dietary lactose (Table 1).

**Table 1:** Summary of studies evaluating the effect of dietary lactose concentration on growth performance of weanling pigs used in the meta-analysis

Study	Weaning age, d	Initial BW, kg	Growth promoters^a^	Duration of feeding, d of age	Dietary CP, %	Lactose level, %	ADFI, g/d	ADG, g/d	G:F
Nessmith et al. [43]	19	5.3	+ (AGP)	19–26	–	0, 20, 40	196, 215, 220	234, 252, 246	1.19, 1.18, 1.15
Nessmith et al. [43]	19	5.3	+ (AGP)	26–33	–	0, 20, 40	310, 301, 300	328, 322, 322	1.06, 1.08, 1.08
Nessmith et al. [60]	10	3.7	+ (AGP)	10–15	–	0, 20, 40	87, 104, 107	109, 119, 125	1.21, 1.16, 1.15
Nessmith et al. [60]	10	3.7	+ (AGP)	15–20	–	0, 20, 40	152, 180, 180	156, 181, 184	1.02, 1.00, 1.03
Tran et al. [54]	20	6.07	+ (AGP)	20–27	22%	0, 20	113, 117	50, 71	0.44, 0.61
Tran et al. [54]	20	6.07	+ (AGP)	27–34	22%	0, 20	340, 390	286, 310	0.84, 0.82
Tran et al. [54]	20	6.07	+ (AGP)	34–48	22%	0, 15	765, 828	545, 567	0.71, 0.68
Tran et al. [54]	20	6.07	+ (AGP)	48–55	22%	0, 5	1044, 1072	717, 719	0.69, 0.67
O′ Doherty et al. [47]	24	7.6	–	24–31	20%	15, 25	242, 239	100, 146	0.41, 0.59
O′ Doherty et al. [47]	24	7.6	–	31–38	20%	15, 25	415, 450	302, 325	0.75, 0.73
O′ Doherty et al. [47]	24	7.6	–	38–49	20%	15, 25	682, 683	438, 388	063, 0.57
O′ Doherty et al. [46]	22, 28	7, 7.7	+ (AGP)	25–32	22%	0, 17.5, 35	209, 223, 224	72, 122, 163	0.34, 0.55, 073
O′ Doherty et al. [46]	22, 28	7, 7.7	+ (AGP)	32–42	22%	0, 17.5, 35	363, 403, 404	180, 281, 265	0.46, 0.66, 0.61
O′ Doherty et al. [46]	22, 28	7, 7.7	+ (AGP)	42–50	22%	0, 17.5, 35	558, 660, 704	267, 424, 478	0.47, 0.64, 0.65
Kim et al. [48]	21	6.94	+ (AGP)	21–28	21%	15, 25	298, 335	247, 289	0.83, 0.86
Kim et al. [48]	28	9.57	+ (AGP)	28–35	21%	15, 25	542, 579	423, 461	0.78, 0.79
Pierce et al. [49]	24	7.1	–	24–31	20%	6.5, 17, 27.5	189, 192, 196	73, 80, 80	0.39, 0.42, 0.41
Pierce et al. [49]	24	7.1	–	31–38	20%	6.5, 17, 27.5	335, 329, 341	175, 224, 212	0.52, 0.68, 0.63
Pierce et al. [49]	24	7.1	–	38–45	20%	6.5, 17, 27.5	512, 511, 567	292, 322, 358	0.61, 0.68, 0.66
Pierce et al. [49]	24	7.1	–	45–52	20%	6.5, 17, 27.5	652, 677, 737	399, 461, 487	0.56, 0.65, 0.63
Pierce et al. [33]	24	6	–	24–31	22%	17, 29.5	254, 323	99, 179	0.39, 0.56
Pierce et al. [33]	24	6	–	31–38	22%	17, 29.5	441, 507	290, 331	0.66, 0.65
Pierce et al. [33]	24	6	–	38–45	22%	17, 29.5	757, 876	452, 527	0.6, 0.61
Mahan and Newton [50]	23	6.5	+ (AGP)	23–37	–	0, 22.5, 35.0	301, 338, 312	243, 278, 258	0.81, 0.82, 0.82
Bertol et al. [51]	21		+ (AGP)	21–35	20%	0, 7, 14, 21	240, 280, 300, 334	142, 186, 191, 219	0.55, 0.65, 0.63, 0.65
Bertol et al. [51]	19	6.3	+ (AGP)	19–26	–	10, 15, 20, 25, 30, 35	190, 207, 202, 214, 225, 194	84, 99, 103, 114, 130, 103	0.42, 0.48, 0.51, 0.53, 0.58, 0.53
Bertol et al. [51]	19	6.3	+ (AGP)	26–33	–	10, 15, 20, 25, 30, 35	401, 412, 423, 428, 421, 409	346, 349, 370, 360, 359,330	0.86, 0.85, 0.88, 0.84, 0.86, 0.83
Bertol et al. [51]	19	6.3	+ (AGP)	26–33	–	7, 12, 17, 22, 27, 32	421, 397, 426, 448, 427, 449	278, 285, 315, 335, 317, 331	0.66, 0.72, 0.74, 0.75, 0.74, 0.74
Bertol et al. [51]	19	6.3	+ (AGP)	33–40	–	7, 12, 17, 22, 27, 32	700, 690, 736, 718, 705, 718	507, 509, 525, 514, 529, 540	0.72, 0.74, 0.71, 0.70, 0.75, 0.75
Bertol et al. [51]	19	6.3	+ (AGP)	40–54	–	0, 5, 10, 15, 20	1095, 1104, 1106, 1124, 1135	657, 685, 678, 695, 686	0.6, 0.62, 0.61, 0.62. 0.60
Molino et al. [58]	21	6.1	+ (AGP)	21–35	21%	0, 4, 8, 12	250, 308, 280, 306	195, 244, 220, 248	0.78, 0.78, 0.78, 0.8
Cromwell et al. [59]	20	6.2	+ (AGP)	30–37	22%	0, 2.5, 5, 7.5, 10	613, 618, 643, 646, 648	467, 467, 503, 505, 506	0.76, 0.76, 0.78, 0.78, 0.78
Cromwell et al. [59]	20	6.2	+ (AGP)	37–44	22%	0, 2.5, 5, 7.5, 10	833, 830, 826, 860, 856	597, 600, 592, 610, 610	0.72, 0.73, 0.73, 0.72, 0.72
Pierce et al. [61]	21	7.6	–	33–45	16%	12.5, 21.5	600, 600	270, 300	0.45, 0.5
Pierce et al. [61]	21	7.6	–	45–54	16%	12.5, 21.5	920, 870	520, 490	0.55, 0.51
Pierce et al. [61]	21	7.6	–	33–45	18.5%	12.5, 21.5	650, 640	400, 380	0.61, 0.59
Pierce et al. [61]	21	7.6	–	45–54	18.5%	12.5, 21.5	1070, 890	620, 590	0.56, 0.66
Pierce et al. [61]	21	7.6	–	33–45	21%	12.5, 21.5	600, 700	400, 400	0.67, 0.59
Pierce et al. [61]	21	7.6	–	45–54	21%	12.5, 21.5	970, 1100	570, 700	0.6, 0.63
O′ Connell et al. [62]	24	6	–	24–31	20%	17, 27.5	190, 212	104, 121	0.55, 0.57
O′ Connell et al. [62]	24	6	–	31–38	20%	17, 27.5	434, 462	364, 394	0.84, 0.85
O′ Connell et al. [62]	24	6	–	38–49	20%	17, 27.5	615, 691	422, 475	0.69, 0.69
O′ Connell et al. [62]	24	6	–	49–57	20%	17, 27.5	870, 883	561, 538	0.65, 0.61

^a^+ indicates the use of in feed antibiotic growth promotors (AGP) and/or pharmacological dose of ZnO (> 1500 ppm) and/or pharmacological dose of CuSO_4_ (> 150 ppm). *ADFI* Average daily feed intake, *ADG* Average daily gain, *BW* Body weight, *CP* Crude protein, *G:F* The ratio of weight gain to feed intake

Models were created for 3 growth phases: d 0–7, d 7–14, and d 14–35 post-wean to reflect the physiological lactase activity of piglets post-wean as well as commercial phase feeding practices [30]. Dietary lactose concentration was used as the sole predictor variable in the models. A total of 12 studies containing 38 experimental diets varying in dietary lactose levels, an average weaning age of 22 d and initial BW of 6.56 kg were used in model development for d 0–7 post-wean. Ten studies with a total of 40 experimental diets, an average start age of 26 d and initial BW of 7.06 kg were used in model development for d 7–14 post-wean. Finally, for d 14–35 post-wean, 9 studies with a total of 54 experimental diets, an average start age of 38 d and initial BW of 10.96 kg were used in model development. In the models, different weaning ages were not included as a covariate because there was not large variation in weaning age amongst studies. There were 2 data points with weaning age of 10 and 4 data points with weaning age of 28, while the rest of the data points (40) had weaning ages from 19 to 24 d. Therefore, more data is needed with an early weaning age and much later weaning ages to use weaning age in the model. The selected data were then subjected to a statistical meta-regression based on a mixed model methodology [63]. Accordingly, different studies were treated as random effects, whereas dietary lactose level was considered a fixed effect. For the predictor variable of dietary lactose levels, the following model was used:

$$ {\mathrm{Y}}_{\mathrm{i}\mathrm{j}}={\mathrm{B}}_0+{\mathrm{B}}_1{\mathrm{X}}_{\mathrm{i}\mathrm{j}}+{\mathrm{B}}_2{{\mathrm{X}}_{\mathrm{i}\mathrm{j}}}^2+{\mathrm{s}}_{\mathrm{i}}+{\mathrm{b}}_{\mathrm{i}}{\mathrm{X}}_{\mathrm{i}\mathrm{j}}+{\mathrm{e}}_{\mathrm{i}\mathrm{j}} $$

where *Y*_ij_ = dependent variable of ADFI, ADG or G:F, *B*_0_ = overall intercept across all studies, *B*_1_ = linear regression coefficient of *Y* on *X* (fixed effect), *B*_2_ = quadratic regression coefficient of *Y* on *X* (fixed effect), *X*_*ij*_ = value of the predictor variable (dietary lactose level), *s*_*i*_ = random effect of study *i*, *b*_*i*_ = random effect of study *i* on the regression coefficient of *Y* on *X* in study *i*, and *e*_*ij*_ = the unexplained residual error. The linear or quadratic model that resulted in a slope different (*P* < 0.10) from 0 was used. The model that had the greater R^2^ and lower RMSE and AIC was used when both linear and quadratic models were different from 0.

Model predicted pig ADFI, ADG and G:F responses to increased dietary lactose levels are presented in Table 2. Pig ADG (*P* < 0.10) and G:F (*P* < 0.01) linearly increased as lactose concentration increased in diets fed to weanling pigs from d 0–7 post-wean. The slope of the models to predict ADFI was not different from 0 and this was most probably due to the large variation in ADFI across studies from d 0–7 post-wean. This may be a result of an immature digestive tract and lack of appetite for solid feed due to large adaptations and high levels of stress in pigs in the first week after weaning. This can potentially be ameliorated by later weaning (e.g. 35 d) and/or the application of a pre-starter diet.

**Table 2:** Meta-regression of growth performance in weanling pigs to changes in different concentrations of dietary lactose

Items	Response variable	Linear R^2^	Linear RMSE	Linear AIC	Linear *P*-value	Quadratic R^2^	Quadratic RMSE	Quadratic AIC	Quadratic *P*-value
d 0–7 post-wean									
ADFI, g/d	Lactose level, %	0.800	66.66	235.5	0.313	0.802	68.85	237.6	0.504
ADG, g/d	Lactose level, %	0.901	48.35	226.5	0.069	0.903	49.63	229.1	0.259
G:F	Lactose level, %	0.995	0.025	15.01	0.001	0.996	0.135	30.8	0.141
d 7–14 post-wean									
ADFI, g/d	Lactose level, %	0.992	13.38	580.0	0.001	0.993	12.69	593.2	0.094
ADG, g/d	Lactose level, %	0.963	24.73	603.3	0.004	0.973	21.86	158.0	0.031
G:F	Lactose level, %	0.887	0.062	37.6	0.435	0.904	0.093	0.058	0.093
d 14–35 post-wean									
ADFI, g/d	Lactose level, %	0.388	167.2	534.6	0.830	0.388	169.4	534.5	0.844
ADG, g/d	Lactose level, %	0.520	98.15	496.3	0.726	0.522	99.32	497.1	0.696
G:F	Lactose level, %	0.705	0.05	−49.2	0.723	0.708	0.051	−33.5	0.514

Pigs begin to increase feed intake 1 week after weaning because the gastrointestinal tract begins to adapt to the solid diets. Accordingly, linear and quadratic models to predict ADFI, ADG and G:F of weanling pigs from d 7–14 post-wean were significantly different from 0. Modeled results indicated that ADG was greatest with an inclusion of 15% lactose, but 30% lactose optimized ADFI. The model also indicated ADG of pigs was reduced when pigs were fed levels of dietary lactose greater than 15% during d 7–14 post-wean. It may be inferred that excessive fermentation of lactose may occur over 15% dietary lactose fed to pigs in d 7–14 post-wean because 30% lactose optimized ADFI, but 15% lactose optimized ADG. Excessive fermentation of lactose may result in osmotic imbalance in the intestinal tract and may lead to diarrhea and reduced ADG of pigs.

There was no linear (*P* > 0.05) or quadratic (*P* > 0.05) response of dietary lactose levels on growth performance of pigs from d 14–35 post-wean. Lactose most probably had a limited effect on performance in this phase due to low lactase activity. The negative ADFI, ADG, and G:F responses to increased levels of dietary lactose, although not significant, indicated that lactose may even be detrimental to growth performance of pigs in d 14–35 post-wean. We hypothesize that this could be due to reduced lactase activity, increased feed intake, and excessive fermentation of lactose as pigs get older.

**Fig. 3:**
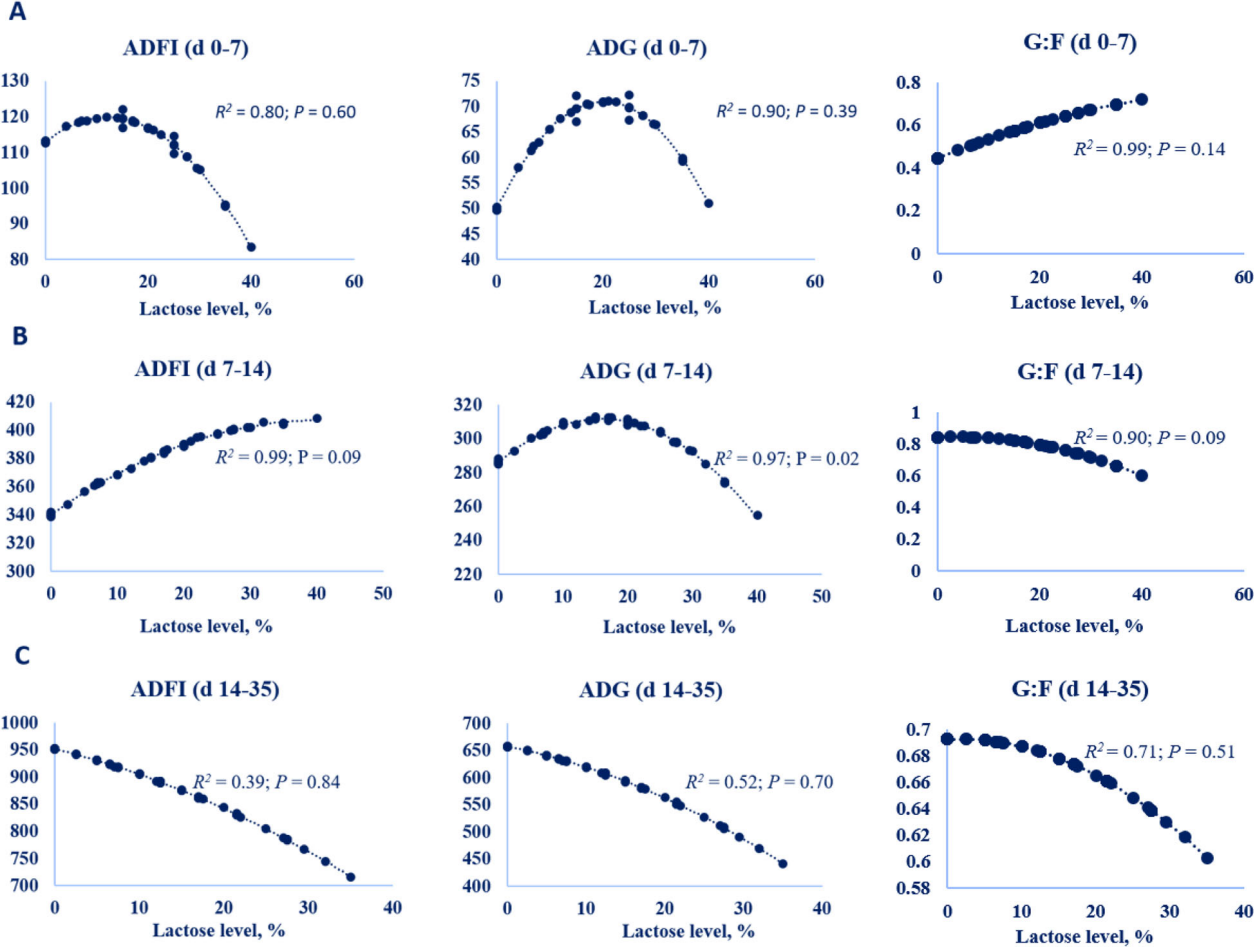
Models developed from literature to determine the response of dietary lactose level on predicted daily feed intake (ADFI, g/d), average daily gain (ADG, g/d) and G:F (ADG: ADFI) of weanling pigs. Meta-analysis was performed using GLM procedure of SAS 9.4. The linear and quadratic equations of ADFI, ADG and G:F were predicted, and only the quadratic equations are presented. **a** the ADFI, ADG or G:F responses of d 0–7 post-wean piglets in relation to dietary lactose level were predicted by using 12 studies (*n* = 38); **b** the ADFI, ADG or G:F responses of d 7–14 post-wean piglets to dietary lactose level were predicted by using 10 studies (*n* = 40); **c** the ADFI, ADG or G:F responses of d 14–35 post-wean piglets to dietary lactose level were predicted by using 9 studies (*n* = 54)

In summary, dietary lactose levels produced different responses on ADFI, ADG and G:F of pigs during different feeding phases, which appear to be associated with the reported lactase activity as pigs get older. In the first week post-wean, there was no limitation of lactose based on responses of ADG and G:F in weanling pigs. This indicates that lactase activity is sufficient to digest high quantities of lactose from d 0–7 post-wean. However, pig ADG decreased when pigs were fed levels of dietary lactose greater than 15% from d 7–14 post-wean. This may be caused by excessive intake and fermentation of lactose due to reduced lactase activity. Therefore, results of the meta-analysis indicate that high lactose levels can be fed to pigs in the early period after weaning, but a decreased inclusion level of lactose should be considered to avoid negative responses of pig ADFI and ADG in later periods. A quadratic regression model was applied (Fig. 3) to determine a potential dietary lactose requirement for weanling pigs based on the literature. The model indicated that the level of dietary lactose to optimize ADG of weanling pigs from d 0–7, 7–14, and 14–35 post-wean was 20%, 15%, and 0, respectively, for piglets with an average initial BW of 6.56 kg and weaned at 22 d of age. The recommendations for pigs from d 0–7 and d 7–14 post-wean from the present meta-analysis are similar to the dietary lactose recommendations provided by Mahan et al. [52] and Cromwell et al. [59]. However, both Mahan et al. [52] and Cromwell et al. [59] have much higher lactose recommendations for pigs from d 14–35 post-wean, whereas our results recommend the addition of no lactose in this later phase of the nursery. Some previous studies reported that the positive effects of dietary lactose supplementation on growth performance of weanling pigs was dependent on the cleanliness of the environment [1, 59] and this could be a reason for the discrepancy in recommendations amongst many other factors.

Therefore, it was hypothesized that pigs fed diets containing antibiotic growth promotors (AGP; antibiotics, pharmacological levels of ZnO, and/or supra-nutritional levels of Cu) would have a cleaner environment and, therefore, a different response to dietary lactose levels. This hypothesis was tested using the same meta-analysis technique in our review. A total of 6 studies containing 54 experimental diets with AGP addition and 4 studies containing 18 experimental diets without AGP were each separately used to develop models to determine the quadratic effect of lactose level on growth performance of pigs during d 0–14 post-wean. Further, for d 14–35 post-wean, 4 studies containing 28 experimental diets with AGP addition and 5 studies containing 26 experimental diets without AGP were each separately used to develop models to determine the quadratic effect of lactose level on growth performance of pigs (Table 1). The model determined that using diets which contained AGP numerically decreased the positive ADFI, ADG and G:F responses of pigs fed greater levels of lactose in diets fed to pigs from d 0–14 and d 14–35 post-wean (Supplementary Figure 1). This may indicate that the prebiotic effect of lactose in weanling pigs may be minimized by the use of AGP. These results are based on the numerical comparison of slopes of the 2 quadratic models (+AGP vs. −AGP) and further interpretation and use of the results are cautioned due to the fact that the slopes of all models were not different (*P* > 0.10) from 0. The observations for no significant responses of dietary lactose levels on pig ADFI and ADG is most probably due to an insufficient number of data points (i. e. references containing AGP versus references without AGP). Therefore, a larger dataset is warranted to further investigate the potential interaction between AGP and the prebiotic role lactose may play in weanling pig nutrition, especially in farms that have poor pig hygiene and health.

## Interactions between lactose and other nutrients or feed additives on growth performance

Interactions have been reported on performance of weanling pigs between lactose level and dietary nutrients or feed additives, such as β-glucanase, organic acids, antibiotics, protein and fermentable dietary fiber. For example, lactose levels above 7.5% in diets fed from d 0–7 post-wean and above 6.3% lactose in diets fed from d 7–14 post-wean resulted in improved ADG, but the combination of an acidifier and high lactose did not further alter growth performance [64]. Lynch et al. [65] found an interaction between crude protein (CP) and lactose concentration that resulted in a positive effect of 23% lactose on performance of piglets (BW of 7.4 kg and weaned at 24 days old) fed diets containing 20% CP compared with pigs fed diets containing 5% lactose and 20% CP, but no positive effects of lactose concentration on growth performance of weanling pigs when fed a 16% CP diet. Furthermore, Pierce et al. [61] indicated growth performance of piglets with an initial age of 35 d and fed a high lactose diet (21.5% vs. 12.5%) was positively related to protein level of diets (16%, 18.5% and 21%). This may be partially explained by the relative effects of high lactose on improved diet palatability and thereby increased ADFI, which resulted in a larger quantity of protein consumed.

The inclusion of 60 mg/kg avilamycin and inulin in a diet with 17.5% lactose fed for 2 weeks post-wean resulted in a proportional improvement in ADG of piglets (initial BW of 6 kg). No positive effects were observed, however, when pigs were fed a diet containing 29.5% lactose, 60 mg/kg avilamycin and inulin [49]. The lack of pig growth performance response to high dietary lactose in these studies may indicate that the addition of antibiotics or dietary fiber may improve intestinal health similar to a high concentration of lactose, resulting in a decreased recommended level of dietary lactose. This result is in support of our interpretation of a limited model created to compare the use of lactose in diets with and without AGP (Supplementary Figure 1).

Weanling pigs (initial BW of 6 kg and 24 d of age) fed a barley-based diet containing 27.5% dietary lactose had decreased ADG compared with piglets fed the diet with 17% lactose in the first week after weaning, but the result of the effect of dietary lactose level on growth performance was reversed when piglets were fed a wheat-based diet [62]. This interaction on performance of pigs between lactose supplementation and cereal type may be explained because the β-glucan in barley may potentially replace a part of the lactose as a prebiotic substrate for fermentation by gastrointestinal microbiota. In a previous study, the inclusion of β-glucanase to a diet containing 17% lactose improved ADG, G:F and nutrient digestibility in piglets with an initial BW of 6.5 kg and weaned at 24 days old, but no positive effects on ADG and G:F were observed when β-glucanase was added to the diet with 27.5% lactose [66]. This observation was most probably caused by the interaction between dietary lactose and β-glucanase, which was associated with a greater digestibility of dietary fiber and production of VFA induced by β-glucanase supplementation, thus minimizing the prebiotic effects of lactose. These results support the use of lactose as a prebiotic for weanling pigs. However, to achieve a consistent and beneficial response, the role of dietary fiber, AGP, and β-glucanase, amongst other factors, and their interactions must be clearly understood and is subject for further investigation.

## Effect of lactose on nutrient digestibility

Increasing dietary lactose can improve nutrient digestibility because it is an easily digestible and/or fermentable component in the intestine of weanling pigs and this results in a greater apparent digestibility of nutrients [47, 67]. Another explanation for the positive effect of lactose on nutrient digestibility could be due to lactic acid and VFA produced by lactose fermentation in the stomach which decreases stomach pH, and thereby improves pepsin activity [45]. A combination of 15% pure lactose and crystalline amino acids added to a weanling pig (initial BW of 6.8 kg and a weaning age of 23 d) diet improved the apparent total tract digestibility (ATTD) of dry matter (DM), gross energy (GE), and nitrogen retention compared with pigs fed a diet containing lactalbumin. Lactalbum was extracted from sow milk and is a primary protein component of whey powder [67]. Furthermore, inclusion of 25% lactose in the diet increased the ATTD of nutrients and energy by weanling pigs (average initial BW of 7.6 kg) compared with pigs fed a diet containing 15% lactose [47]. The inclusion of 1.5% inulin improved energy digestibility by pigs (average initial BW of 6.0 kg) fed a diet with 15% lactose, but did not affect the digestibility by pigs fed the high (29.5%) lactose diet [49]. The negative response of high lactose level with inulin supplementation may be caused by excessive fermentation of lactose and inulin by gut microbiota, resulting in abnormal intestinal osmotic balance. Including 27.5% dietary lactose decreased the nutrient digestibility by weanling pigs fed a barley-based diet compared with a barley-based diet supplemented with only 17% lactose. In contrast, pigs fed a wheat-based diet saw in no difference in nutrient digestibility whether or not the diets contained 27.5% or 17% lactose [62], which may also be caused by more fiber fermentation provided by barley than the wheat-based diet. These results indicate that the effect of dietary lactose on nutrient digestibility may be associated with dietary composition and most probably related to dietary fiber and microbial fermentation potential. Bach Knudsen [68] compared the ileal digestibility of β-glucan by 40–50 kg growing pigs fed diets based on rolled oats, oat groats, oat flour, or oat bran and contained either a high lactose (12 to 38 g lactose/kg DM) or low lactose content (0 to 1 g lactose/kg DM). Results indicated that the digestibility of β-glucan in the ileum was 64% in diets with low lactose and 27% in diets containing high lactose, which suggested that lactose was preferentially fermented by gastrointestinal microbiota compared to β-glucan when there was most likely very little intestinal lactase activity in pigs of 40–50 kg. This study provides further evidence that lactose is preferentially fermented by gastrointestinal microbiota compared with other dietary fibers and that this interaction must be accounted for in order to achieve a prebiotic effect with dietary lactose. A prebiotic effect of lactose is when dietary lactose is not digested, rather it is used as a substrate for fermentation by microbes and this elicits a change in microbial populations and metabolites that improve the health of the pig.

## Effect of lactose on intestinal morphology

Pierce et al. [69] reported no difference in villus height and crypt depth of intestinal segments in weanling piglets (mean initial BW of 7.8 and average 21 d of age) fed diets with 15% or 33% lactose, although piglets fed the high lactose diet did have a reduced cecum and colon pH. Furthermore, in the same study, the inclusion of 1.5% inulin into the 15% lactose diet resulted in increased villus height, but inulin added to the diet with 33% lactose had no effect on pig gut morphology. This indicates that an interaction between dietary lactose level and inulin (i.e., fermentable substrates) on intestinal morphology of weanling piglets exists. There were also no differences in intestinal morphology on d 0, 3, and 10 post-wean of piglets fed diets containing either 20% glucose, lactose or starch [1]. This may partly be due to the fact that these pigs were weaned later (28 d of age) and with a heavier BW (8 kg) than those in most other lactose studies. The addition of 0 or 12% lactose in the diet did not affect villous height, crypt depth or villous: crypt ratio of weanling pigs in the first 2 weeks post-wean [58]. This report is not in agreement with the previous study that reported a positive effect of lactose supplementation on intestinal villus height and capacity for nutrient absorption in weanling pigs [51]. Acosta et al. [70] also reported an increased villous height when weanling piglets (average initial BW of 5.2 kg and 21 d of age) were fed a diet containing 15% lactose compared with a diet containing 3% lactose. Also, a diet with a high lactose to protein ratio improved the villous height of the proximal small intestine in weanling piglets (26 d of age) compared with a low lactose to protein ratio diet [71]. These results indicate that the digestibility of dietary lactose, rather than protein, could provide more digestible energy or a suitable micro-environment for the development of intestinal epithelial cells. Overall, there have been inconsistent effects of dietary lactose on intestinal morphology in weanling pigs. The inconsistency most likely is associated with variations in dietary lactose level, lactase activity of weanling pigs, and dietary composition. Further studies should be conducted to explore the impact of energy supplied versus the prebiotic response derived from lactose digestion by lactase versus fermentation by gastrointestinal microbiota on intestinal morphology and the health of weanling pigs.

## Effect of lactose on gastrointestinal microbiota

Dietary lactose has been shown to have prebiotic effects on microbiota in the gastrointestinal tract of weanling piglets [72]. This is because it may be partially fermented by microbiota to lactic acid and VFA to provide gastric and colonic acidity [68]. Lactic acid and VFA can reduce stomach pH and create a less favorable environment for pathogens and suppress the growth of *E. coli*, thereby improving the gastrointestinal health of the host [73]. Furthermore, a high concentration of dietary lactose (20%) increased the relative abundance of lactobacilli and reduced *E. coli* in the large intestine of piglets in the first week post-wean [54]. The inclusion of lactose in diets for weanling pigs increased the population of total bacteria in the colon and *Lactobacillus* abundance in the ileum and colon per gram of digesta content [74]. The inclusion of either 25% or 15% lactose decreased the counts of *E. coli* in the feces of piglets after weaning [47]. The inclusion of lactose in the diet resulted in a greater diversity and abundance of the beneficial bacteria in the intestine of weanling pigs in the first 2 weeks post-wean, and the *Lactobacillus* richness increased with the addition of 8% lactose, although the population of total bacteria decreased when piglets were fed 12% lactose [64]. In addition, the supplementation of *Bifidobacterium* increased lactose fermentation and lactic acid production, but not acetate, helping to regulate the microbial composition in the gastrointestinal tract of piglets [75]. Overall, our findings in this review indicate that dietary lactose has a prebiotic effect on the gastrointestinal microbiota. This mode of action of lactose must be further explored to enhance weanling pig gastrointestinal health in light of new legislation and consumer demands limiting the use of AGP, pharmacological levels of ZnO, and supra-nutritional levels of Cu. However, a high lactose supplementation and/or intake may result in excessive fermentation of lactose and this could be detrimental to growth performance as hypothesized from our meta-analysis as well as increase risk of post-weaning diarrhea when weaning stress and herd hygiene and health are not good. Thus, the prebiotic action of lactose fed to weanling pigs needs to be further explored.

## Lactose in growing-finishing pig diets

Few studies have reported effects of dietary lactose fed to growing-finishing pigs from 25 to 130 kg BW. This is mostly because adult pigs have a high capacity for digesting other carbohydrates which are typically less costly than lactose [76]. A previous study reported a reduced ATTD of nutrients in adult pigs offered a diet containing 16% lactofeed (Volac International Ltd., Orwel, Royston, UK; 95.5% dry matter, 12.5% protein, 5.0% oil, 9.0% ash, 1.0% fiber, 70% lactose and a pH of 6.5–7) for 3 weeks [76]. This result suggests that lactose is not well digested by older pigs due to low lactase activity, and is most probably fermented, which results in a reduced energetic contribution of lactose to the pig. It is hypothesized that excessive intake of lactose may result in a possible overloading of the lactase digestion system which may result in excessive quantities of rapidly fermentable carbohydrate entering the cecum and colon which may exceed the fermentative capacity of the pig [39]. Visscher et al. demonstrated an increased lactic acid concentration in the cecum of 130 kg pigs fed a diet containing 16.9% whey powder for 4 weeks and this coincided with a reduction in *Lawsonia intracellularis* in the cecum. This indicates that lactose was not digested by the fattening pig, rather it was fermented. Furthermore, the increased lactic acid coincided with a reduction in *Lawsonia intracellularis*, which is a pathogen that can cause intestinal damage and disease in swine [38]. This study, therefore, further demonstrates the potential prebiotic effect from lactose fermentation. Furthermore, a high nutrient digestibility and nitrogen retention by grow-finish pigs fed a diet containing 12% lactofeed was observed which indicated that this level of lactose is optimal in terms of improving dietary protein utilization and reducing nitrogen excretion, but had no positive effects on ADFI and ADG of pigs [76]. However, there was no further increase in lactobacilli concentrations above 4% lactofeed in diets, indicating that small quantities of dietary lactose are sufficient to alter conditions in the distal small intestine and large intestine due to low lactase activity in growing-finishing pigs. This also further corroborates our hypothesis that lactase activity is independent of the levels of dietary lactose fed as well as the duration of lactose feeding [5] and that there is a balance between optimal fermentation of lactose and excessive fermentation of lactose that positively, or negatively, effect gastrointestinal health.

## Hypothesized modes of action of dietary lactose in weanling pigs

**Fig. 4:**
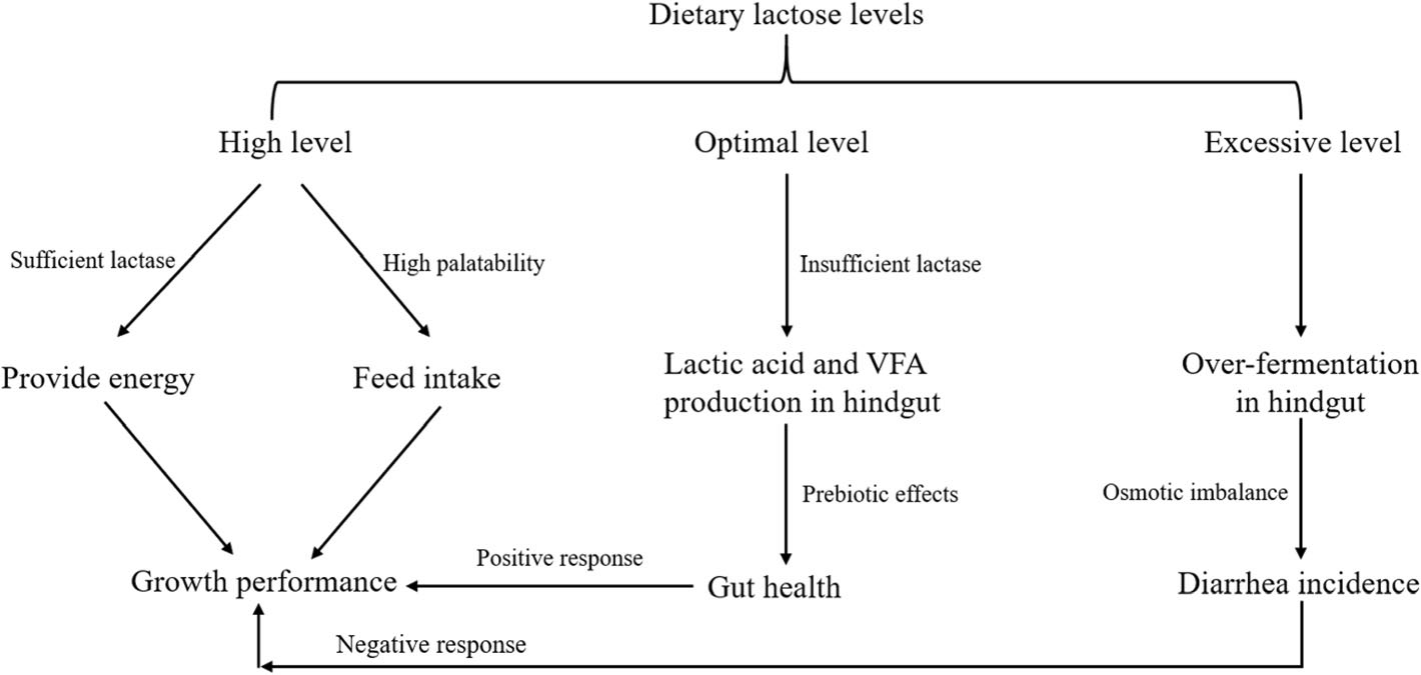
Proposed mode of actions of dietary lactose levels in relation to growth performance, gut health, and diarrhea in weanling pigs

Based on the comprehensive evidence demonstrated above, the proposed mode of action of dietary lactose in relation to growth performance, gut health, and risk of diarrhea in weanling pigs was demonstrated in this review (Fig. 4). The results of the meta-analysis showed no limitation of dietary lactose concentration on pig growth performance from d 0–7 post-wean. These results, taken together with the other effects of lactose reported in the literature, indicate that dietary lactose stimulates feed intake due to a high palatability and improves growth performance due to high lactase activity in the early post-wean period.

Secondly, dietary lactose can be fermented by gut microbiota to produce lactic acid and VFA. Therefore, a slightly higher, but optimal level of dietary lactose will provide lactose in quantities greater than lactase can sufficiently digest thereby making lactose a rapidly fermentable carbohydrate source which has the prebiotic effect on the microflora promoting lactic acid and VFA production which stimulates gut health. This hypothesized mode of action is also supported from the meta-analysis because pig ADG was reduced when dietary lactose level exceeded 15% in diets fed from d 7–14 post-wean, but pig ADFI continued to gradually increase up to 30% lactose inclusion in the diet. Furthermore, a negative response to dietary lactose on pig ADG and ADFI from d 14–35 post-wean was observed in the models based on the meta-analysis. Taken together, an excessive level of lactose fed from d 14–35 post-wean may induce an osmotic imbalance due to limited lactase activity and excessive lactose fermentation and this may lead to diarrhea. These different mechanisms of action of dietary lactose in weanling pig nutrition are only our hypothesis based on models from the meta-analysis and the normal physiology of pigs, such as a reduced lactase activity and dynamic gut microbiota community as pigs get older. To our knowledge these hypotheses have not been directly tested because it is difficult to quantify lactose in intestinal digesta using current laboratory techniques. No studies have reported how much lactose is digested by endogenous lactase activity and how much lactose is fermented to exert prebiotic effects or lead to diarrhea when weanling pigs are fed different amounts of lactose.

## Lactose sources and equivalents in diets

There are many sources of lactose that are used in weanling pig diets such as pure lactose, dry whey powder (both acid and sweet), whey permeate, deproteinized whey, and milk products such as skimmed milk powder, which show different impacts on growth performance on weanling pigs. A previous study reported pigs weaned at 14 d of age and BW of 4.4 kg fed a diet containing 20% dry whey powder had greater growth performance compared with pigs fed other lactose sources because of the high quality of protein and palatability [60]. However, Delgado et al. [77] indicated pure lactose or whey permeate could replace dry whey powder to maintain the same level of dietary lactose without any negative effect on growth performance of weanling pigs. The inconsistent response observed when using lactose sources which contain protein (i.e. whey powder) may be due to heat damage of the protein and sugar present in the lactose source [78]. The majority of lactose sources used in pig diets go through a drying step in their production [79]. This drying step, along with the high concentration of protein and reducing sugars, make these products highly susceptible to Maillard reactions [78]. On the one hand, previous studies have reported that Maillard reaction products exert prebiotic effects on host health such as antibacterial and antioxidant functions [80, 81]. On the other hand, the Maillard reaction results in a complex that renders the protein, most notably lysine, and sugar unavailable to the animal, thereby decreasing nutrient (lysine and energy) availability, and, thus, results in a loss of performance [82]. The Maillard reaction may explain some inconsistencies in growth performance of pigs fed different concentrations of lactose reported in the literature. Thus, the Maillard reaction should be considered in future studies using lactose sources which contain protein and reducing sugars. The potential of the Maillard reaction was not accounted for in the present meta-analysis because it was not reported in the studies and the majority of the studies fed mash diets where pure lactose was used to increase lactose concentration in the diet. Therefore, pig growth performance responses from different lactose levels was most likely not influenced by the existence of Maillard reaction products in our meta-analysis.

In most nursery pig production systems, it is expensive to provide a diet containing a high level of lactose as recommended in this review. Thus, it is crucial to optimize feed formulations of weanling pig diets by choosing suitable lactose sources and/or lactose equivalents that may partially replace the hypothesized modes of action of lactose. From this review, it was suggested that the modes of action of lactose to improve growth performance of pigs may be partially attributed to its’ high palatability to increase feed intake, high digestibility to provide energy, and prebiotic effects to improve gut health. Many different ingredients, additives, and nutrients can be used to replace at least one of the hypothesized modes of action of lactose in weanling pig nutrition. These are so called “lactose equivalents”. For example, dietary fiber, resistant starch and non-digestible oligosaccharides exert prebiotic effects on gut microbiota and health of weanling pigs [83]. Some sugars, like dextrose, sucrose, molasses, and extruded starch can be used to increase ADFI and ADG of weanling pigs because they are an easily digestible energy source and also have a high palatability [84, 85]. Previous reports have indicated that supplementation of dietary fiber, dextrose, sucrose, and other low or non-lactose containing milk by-products can be used to partially replace lactose without any negative effect on growth performance of weanling piglets [26, 86]. Mavromichalis et al. [86] showed a combination of sucrose and molasses could effectively replace 50% and 100% of lactose in diets fed to weanling pigs with average initial BW of 4.6 kg due to the high palatability and digestibility of sucrose and molasses. Guo et al. [87] demonstrated a candy coproduct from chocolate as an energy source used to substitute up to 45% of whey permeate had no negative impact on growth performance of weanling pigs. Overall, it is difficult to completely replace dietary lactose due to a high activity of lactase immediately at weaning and its different mechanisms of action in young pigs. In addition, it should be noted that impacts of dietary lactose on subsequent growth performance of pigs is still unknown when pigs have been fed optimal quantities of lactose in the nursery period. Therefore, the potential carry-over effects of lactose and lactose equivalents on performance of growing-finishing pigs should also be considered when the replacement efficiency of lactose equivalents for weanling pigs are studied.

## Conclusions

Dietary lactose plays a crucial role in improving performance and gastrointestinal environment and health of newly weaned pigs. The positive effect of dietary lactose on the growth performance of weanling pigs is most evident in the first 2 weeks after weaning. This coincides with physiological endogenous lactase activity over time, which is independent of dietary lactose level and duration of lactose ingestion. No significant responses, even negative responses, of dietary lactose levels on growth performance and physiological function of pigs after d 14 post-wean may be associated with a reduced lactase activity and excessive fermentation of lactose in pig intestine, resulting in abnormal intestinal permeability. It is recommended that dietary concentrations of lactose should be 20%, 15%, and 0 for piglets from d 0–7, d 7–14, and d 14–35 post-wean, respectively, based on our meta-analysis. In most situations, the cost of lactose limits its use and commercial nursery pig diets typically do not reach these recommended levels. This is because other ingredients, additives and nutrients can potentially replace lactose. We hypothesized the potential mechanisms of action of lactose fed to weanling pigs to be attributed to the high palatability, high digestibility, and prebiotic effects of lactose to better understand how to use lactose equivalents in the diet. The findings described in this review indicated that dextrose, sucrose, dietary fiber, prebiotics, and milk by-products can partially or completely replace lactose in weanling pig diets. Furthermore, responses to lactose in weanling pigs are influenced by other nutrients, feed additives and AGP supplementation in diets. Therefore, it is also important and necessary to evaluate interactions between lactose and other nutrients or feed additives.

## Supplementary Information

**Additional file 1: Supplementary Figure 1.** Meta-analysis for the quadratic response of dietary lactose level on average daily feed intake (ADFI, g/d), average daily gain (ADG, g/d) and G:F (ADG:ADFI) of weanling pigs. Meta-analysis was performed using GLM procedure of SAS 9.4. A total of 7 studies containing 54 experimental diets with antibiotic growth promoter (AGP) addition and 4 studies containing 18 experimental diets without AGP were used to develop models to predict the response of lactose level on growth performance of pigs between d 0–14 post-wean. A total of 5 studies containing 28 experimental diets with AGP addition and 5 studies containing 26 experimental diets without AGP were used to develop models to predict the response of lactose level on growth performance of pigs between d 14–35 post-wean.

## Data Availability

Please contact author for data requests.

## Abbreviations

ADFI: Average daily feed intake
ADG: Average daily gain
AGP: Antibiotic growth promoters
ATTD: Apparent total tract digestibility
DM: Dry matter
GE: Gross energy
VFA: Volatile fatty acid
