# Supplementary material for: The role of lactose in weanling pig nutrition: a literature and meta-analysis review
Source: J Anim Sci Biotechnol. 2021 Jan 11;12:10. doi: 10.1186/s40104-020-00522-6 (PMC7798279; doi:10.1186/s40104-020-00522-6)
Supplement: Supplementary file 1 — Additional file 1: Supplementary Figure 1. Meta-analysis for the quadratic response of dietary lactose level on average daily feed intake (ADFI, g/d), average daily gain (ADG, g/d) and G:F (ADG:ADFI) of weanling pigs. Meta-analysis was performed using GLM procedure of SAS 9.4. A total of 7 studies containing 54 experimental diets with antibiotic growth promoter (AGP) addition and 4 studies containing 18 experimental diets without AGP were used to develop models to predict the response of lactose level on growth performance of pigs between d 0–14 post-wean. A total of 5 studies containing 28 experimental diets with AGP addition and 5 studies containing 26 experimental diets without AGP were used to develop models to predict the response of lactose level on growth performance of pigs between d 14–35 post-wean. [file 40104_2020_522_MOESM1_ESM.docx]

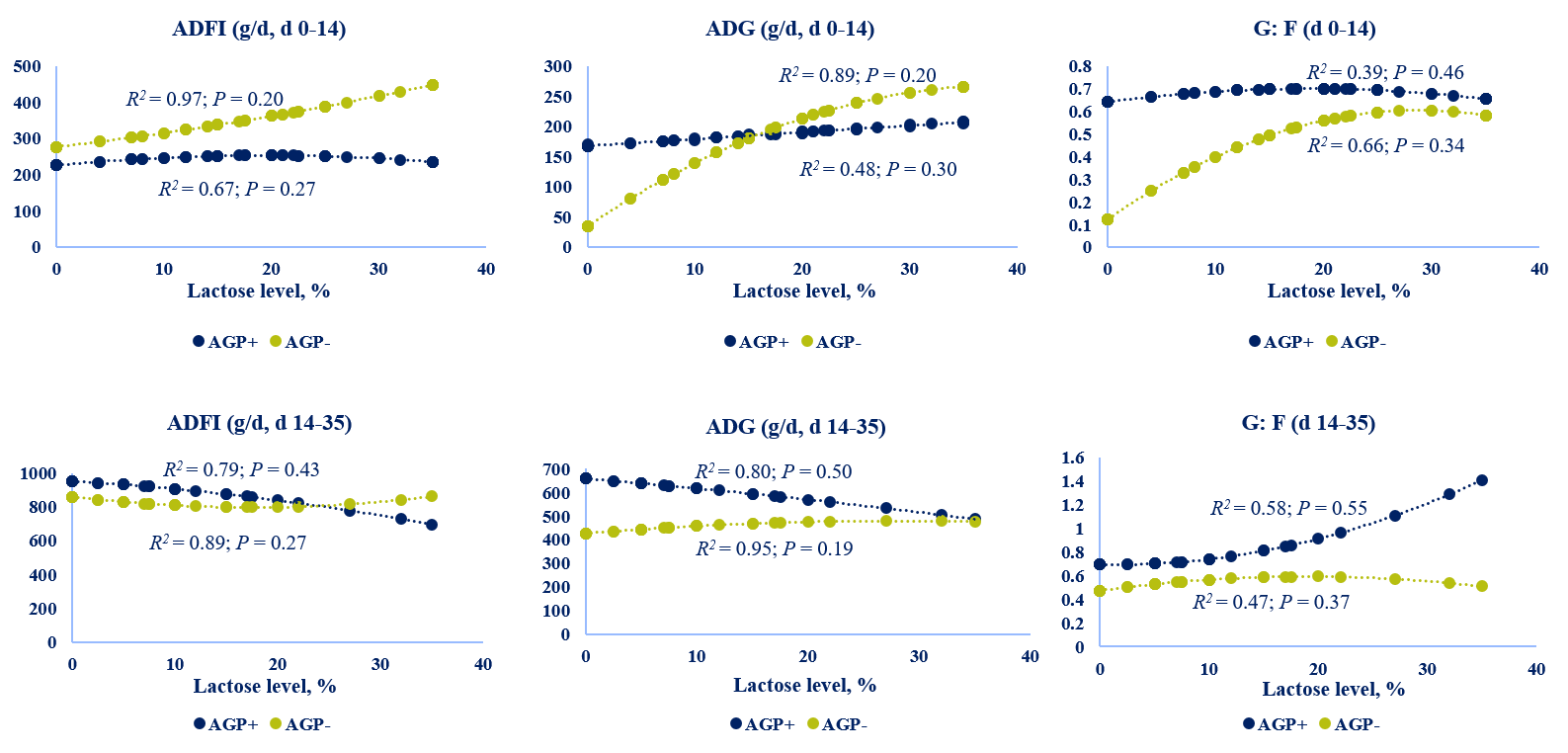
**Supplementary Figure 1** Meta-analysis for the quadratic response of dietary lactose level on average daily feed intake (ADFI, g/d), average daily gain (ADG, g/d) and G: F (ADG: ADFI) of weanling pigs. Meta-analysis was performed using GLM procedure of SAS 9.4. A total of 7 studies containing 54 experimental diets with antibiotic growth promoter (AGP) addition and 4 studies containing 18 experimental diets without AGP were used to develop models to predict the response of lactose level on growth performance of pigs between d 0-14 post-wean. A total of 5 studies containing 28 experimental diets with AGP addition and 5 studies containing 26 experimental diets without AGP were used to develop models to predict the response of lactose level on growth performance of pigs between d 14-35 post-wean.
